# Mechanism of Qianlie Zhitong Formula Against Chronic Prostatitis Based on Proteomics and Metabolomics

**DOI:** 10.1111/cbdd.70401

**Published:** 2026-10-19

**Authors:** Lidan Dong, Linhan Chang, Caijia Song, Jialin Yan, Tiantong Shen, Zhen Ding, Yunyu Liu, Chenglong Zheng, Yanfei Zheng, Qi Wang

**Affiliations:** ^1^ WANG Qi Academy National Institute of TCM Constitution and Preventive Medicine, Beijing University of Chinese Medicine Beijing China; ^2^ Dongzhimen Hospital, Beijing University of Chinese Medicine Beijing China; ^3^ Beijing University of Chinese Medicine Beijing China; ^4^ School of Chinese Materia Medica Beijing University of Chinese Medicine Beijing China

**Keywords:** chronic prostatitis, ferroptosis, metabolomics, proteomics, Qianlie Zhitong formula

## Abstract

Chronic prostatitis/chronic pelvic pain syndrome (CP/CPPS) is a prevalent pelvic pain disorder in men, yet effective pharmacotherapies remain limited. Emerging evidence suggests that oxidative stress and dysregulated iron metabolism contribute to the pathogenesis of CP/CPPS, with ferroptosis increasingly implicated in CP/CPPS‐related pathology. Qianlie Zhitong Formula (QLZTF) has shown therapeutic potential in treating CP/CPPS; however, its underlying mechanisms remain incompletely understood. This study integrated proteomics, metabolomics, and in vivo experiments to investigate the therapeutic mechanisms of QLZTF. A carrageenan‐induced rat model was established, and rats were randomly assigned to five groups: control, model, Qianliekang, low‐dose QLZTF, and high‐dose QLZTF. QLZTF significantly reduced inflammatory cytokines (TNF‐α, IL‐6, and IL‐1β), alleviated pain responses, and ameliorated prostate tissue injury. Proteomic analysis identified BCAT2, ALDH1B1, and ALDH2 as significantly upregulated proteins closely associated with ferroptosis. KEGG enrichment analysis of both proteomic and metabolomic data revealed that pantothenate and CoA biosynthesis and branched‐chain amino acid (BCAA) degradation pathways were significantly enriched. Integrated analysis indicated that QLZTF synergistically regulates these pathways, activates the BCAT2/ALDH axis, and inhibits ferroptosis, thereby alleviating inflammation, pain, and fibrosis in CP/CPPS. This study provides the first multiomics evidence for the anti‐ferroptotic mechanism of QLZTF.

## Graphical abstract

QLZTF blood‐absorbed bioactive components (hydroxysafflor yellow A, saikosaponin A and D) activate two core metabolic pathways (pantothenic acid and CoA biosynthesis, BCAA degradation) in prostatic epithelial cells. This suppresses ferroptosis and the secretion of pro‐inflammatory IL‐17/IFN‐γ, thereby ameliorating prostatic edema, interstitial fibrosis, pelvic hyperalgesia and glandular inflammatory injury in CP/CPPS.

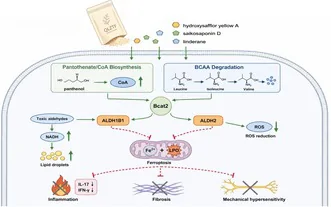

## 1 Introduction

Prostatitis is an inflammatory syndrome of the male genitourinary system (Song et al. 2026). Chronic non‐bacterial prostatitis/chronic pelvic pain syndrome (CP/CPPS), corresponding mainly to NIH Category III prostatitis, is the most prevalent subtype and accounts for approximately 90%–95% of prostatitis cases (Song et al. 2026; Lai et al. 2025a). Its main manifestations include pelvic pain, urinary symptoms, and sexual dysfunction, and its chronic and recurrent course often causes substantial psychological distress and impaired quality of life (Song et al. 2026; Lai et al. 2025a; Alshahrani et al. 2025). Although NSAIDs, neuromodulators, α‐blockers, antibiotics, phytotherapeutic agents, pelvic floor approaches, and multimodal strategies are used clinically, the pathogenesis and optimal treatment of CP/CPPS remain incompletely understood (Song et al. 2026; Qin et al. 2022; Franco et al. 2020; Lai et al. 2025b). In addition, the blood‐prostate barrier and the heterogeneous inflammatory microenvironment may limit drug delivery and therapeutic efficacy (Song et al. 2026). These limitations highlight the need for new therapeutic strategies and systematic mechanistic studies of candidate interventions.

Qianlie Zhitong Formula (QLZTF) is used in this study as an empirical decoction for CP/CPPS under the “principal‐disease principal‐decoction” framework. QLZTF was developed based on the therapeutic principles of Fuyuan Huoxue Decoction (FYHXD), a classical decoction traditionally attributed to Li Dongyuan and recorded in Yixue Faming, which has been used for traumatic injury‐related blood stasis and pain (Wu 2011; Xie et al. 2018). FYHXD embodies blood‐activating, stasis‐resolving, liver‐soothing, collateral‐dredging, and pain‐relieving strategies, which are consistent with the therapeutic rationale of QLZTF. Modern clinical observations suggest that modified FYHXD has satisfactory therapeutic effects for NIH‐III prostatitis and chronic non‐bacterial prostatitis, including improvements in NIH‐CPSI scores, urinary symptoms, quality of life, and inflammatory/immune mediators in expressed prostatic secretions, such as IL‐8, IL‐10, and TGF‐β1 (Wu 2011; Xie et al. 2018). However, the proteomic and metabolomic mechanisms underlying QLZTF intervention in CP/CPPS remain insufficiently characterized. Recent mechanistic studies indicate that CP/CPPS is associated with inflammation, oxidative stress, metabolic perturbation, and ferroptosis‐related pathological changes (Ma et al. 2025; Zhang et al. 2025; Lin et al. 2022). We therefore hypothesized that QLZTF could modulate ferroptosis‐related inflammatory and metabolic networks in CP/CPPS. To test this hypothesis, this study combined UPLC–MS‐based component analysis, proteomic profiling, untargeted metabolomic profiling, and validation of key ferroptosis‐related molecules to explore the potential mechanisms underlying the therapeutic effects of QLZTF in CP/CPPS.

## 2 Materials and Methods

### 2.1 Materials

Carrageenan (#C121014) was obtained from Shanghai Aladdin Biochemical Technology Co. Ltd. (Shanghai, China). Qianliekang (NMPA Approval No. Z33020303) was obtained from Zhejiang Kangnobe Pharmaceutical Co. Ltd. (Zhejiang, China). Quantitative measurement of pro‐inflammatory cytokines TNF‐α (catalog No. RTA00), IL‐6 (catalog No. R6000B), and IL‐1β (catalog No. RLB00) was done in the prostate tissue of both CNP and treated groups using commercial ELISA assay kits (R&D Systems (Minneapolis, MN, USA)), according to the manufacturer's instruction. The samples and standards were all run in quadruplicate and the data were averaged. The results are expressed as pg/mL.

### 2.2 Experimental Preparation of Qianlie Zhitong Formula

The Qianlie Zhitong Formula is a proprietary traditional Chinese medicine preparation developed for clinical use in the treatment of chronic prostatitis. Due to proprietary constraints, the exact proportions of the individual components within this formula cannot be disclosed, under Approval No. (Q2203002). Detailed decoction information may be obtained from the corresponding author upon reasonable request, subject to appropriate confidentiality agreements. After being processed into granules, the Qianlie Zhitong Formula should be stored under the following conditions: in a cool place (typically room temperature between 20°C–25°C), in a tightly sealed container, protected from light exposure, and kept ready for use.

### 2.3 Animals and Treatment

Forty male Sprague–Dawley rats (180–200 g) were purchased from Sibeifu Biotechnology Co. Ltd. All experimental procedures were approved by the Institutional Animal Care and Use Committee of Beijing University of Chinese Medicine (approval no. BUCM−4‐2,022,012,102‐1056). After a 7‐day acclimation period, 40 rats were randomly divided into five groups: control group (deionized water 10 mL/kg·d, *n* = 8), model group (deionized water 10 mL/kg·d, *n* = 8), Qianliekang group (dosage: 0.5 g/kg·d, *n* = 8), Qianlie Zhitong Formula low‐dose group (lot number. Q2203002, dosage: 1.7 g/kg·d, *n* = 8) and Qianlie Zhitong Formula high‐dose group (6.8 g/kg·d, *n* = 8).

A rat model of chronic prostatitis was established. Anesthesia was induced by intraperitoneal injection of sodium pentobarbital. Except for the sham‐operated group, all groups received intraprostatic injection of 50 μL of 1% carrageenan solution into each bilateral ventral prostate lobe using a microsyringe (100 μL in total). A chronic prostatitis animal model was thus established; the sham‐operated group received an equal volume of saline. Rats were housed in the animal facility of the Yifu Research Building, Hepingjie Campus, Beijing University of Chinese Medicine under controlled conditions: temperature 20°C–23°C, relative humidity 40%–60%, and free access to food and water. After 4 weeks, the rats were euthanized by cervical dislocation. Immediately after euthanasia, blood and tissue samples were collected. A portion of the tissues was fixed in 4% paraformaldehyde for subsequent histopathological analysis, while the remaining tissues and blood samples were rapidly frozen in liquid nitrogen and then stored at −80°C for further molecular biological assays.

### 2.4 Measurement of Pain Threshold

On days 0 and 28 after carrageenan‐induced modeling, mechanical pain thresholds in the perineal region were measured in each group of rats. Rats were placed individually in a transparent plexiglass box with a metal mesh floor (a minimum 30‐min acclimation period was given before testing). Von Frey filaments (0.4 g, 1 g, and 4 g, applied in ascending order of strength) were used to assess pain sensitivity. Each filament was applied five times, with each stimulus lasting 1–2 s and an interstimulus interval of 2 min. Stimulation was restricted to the lower abdominal area near the prostate, and care was taken to stimulate different sites within this region to avoid desensitization. The following three behaviors were considered positive pain responses to Von Frey filament stimulation: (1) vigorous abdominal contraction; (2) immediate licking or scratching of the stimulated site; (3) jumping. The pain response rate was calculated as the percentage of positive pain responses out of the total number of stimulations (Peng et al. 2021).

### 2.5 Metabolomics Analysis

#### 2.5.1 Metabolite Extraction

Rat prostate tissue samples were slowly thawed at 4°C. An appropriate amount of each sample was taken, and precooled methanol/acetonitrile/water solution (2:2:1, v/v/v) was added, followed by vortex mixing. The mixture was ultrasonicated at low temperature for 30 min, then allowed to stand at −20°C for 10 min, and centrifuged at 14,000 g and 4°C for 20 min. The supernatant was collected and dried under vacuum. For mass spectrometry analysis, the residue was reconstituted in 100 μL of acetonitrile/water solution (acetonitrile: water = 1:1, v/v), vortexed, centrifuged at 14,000 g and 4°C for 15 min, and the supernatant was injected for analysis.

#### 2.5.2 Chromatography‐Mass Spectrometry Analysis

##### 2.5.2.1 Chromatography conditions

Separation was performed using a Vanquish LC ultra‐high performance liquid chromatography (UHPLC) system equipped with a HILIC column. The column temperature was 25°C, flow rate 0.5 mL/min, and injection volume 2 μL. The mobile phase consisted of A: water +25 mM ammonium acetate +25 mM ammonium hydroxide, and B: acetonitrile. The gradient elution program was as follows: 0–0.5 min, 95% B; 0.5–5 min, B linearly decreased from 95% to 65%; 5–5.5 min, B linearly decreased from 65% to 40%; 5.5–6 min, B linearly increased from 40% to 95%; 6–8 min, B was maintained at 95%. Throughout the analysis, samples were kept in an autosampler at 4°C. To avoid fluctuations in instrument detection signals, samples were analyzed consecutively in a randomized order. Quality control (QC) samples were inserted into the sample queue to monitor and evaluate system stability and the reliability of experimental data.

##### 2.5.2.2 Orbitrap Exploris 480 mass spectrometry conditions

After separation by the Vanquish LC UHPLC system, the samples were analyzed using an Orbitrap Exploris 480 mass spectrometer (Thermo) with electrospray ionization (ESI) in both positive and negative ion modes. The ESI source and mass spectrometry parameters were set as follows: nebulizer gas (Gas1): 50, auxiliary heating gas (Gas2): 2, ion source temperature: 350°C, spray voltage (ISVF): 3500 V in positive ion mode and 2800 V in negative ion mode. MS1 scan range: 70–1200 Da, resolution: 60,000, accumulation time: 100 ms. For MS2, a segmented acquisition method was used with a scan range of 70–1200 Da, resolution: 60,000, accumulation time: 100 ms, and dynamic exclusion time: 4 s.

#### 2.5.3 Data Processing

Raw data were converted to .mzXML format using ProteoWizard, followed by peak alignment, retention time correction, and peak area extraction using XCMS software. The data extracted by XCMS were subjected to metabolite structure identification and data preprocessing (missing value filtering: removal of ion peaks with > 50% missing values; missing value imputation: KNN imputation; data filtering: removal of features with RSD > 50%). Subsequently, experimental data quality assessment (six quality control metrics) was performed, followed by data analysis.

### 2.6 Proteomics Analysis

#### 2.6.1 Protein Extraction

Rat prostate tissue samples were homogenized using an MP FastPrep‐24 automated homogenizer (24 × 2, 6.0 M/S, 60 s, twice). SDT lysis buffer (4% SDS, 100 mM Tris–HCl, pH 7.6) was added to the homogenate. The mixture was sonicated, boiled for 15 min, and centrifuged at 14,000 g for 40 min. The supernatant was collected and quantified using a BCA Protein Assay Kit. For each sample, 15 μg of protein was mixed with 5 × loading buffer, boiled for 5 min, and separated on a 4%–20% SDS‐PAGE gel at 180 V for 45 min. Protein bands were visualized by Coomassie Blue R‐250 staining. Equal aliquots of all samples were pooled as a quality control sample.

#### 2.6.2 Protease Digestion and Peptide Preparation

DTT (at a final concentration of 40 mM) was added to each sample and mixed at 600 rpm for 1.5 h (37°C). After the samples cooled to room temperature, IAA was added to the mixture at a final concentration of 20 mM to block reduced cysteine residues, and the samples were incubated for 30 min in the dark. Next, the samples were transferred to the filters (Microcon units, 10 kDa). The filters were washed with 100 μL UA buffer three times and then 100 μL 25 mM NH4HCO3 buffer twice. Finally, trypsin was added to the samples (the trypsin: protein (wt/wt) ratio was 1:50) and incubated at 37°C for 15–18 h (overnight), and the resulting peptides were collected as a filtrate. The peptides of each sample were desalted on C18 Cartridges (Empore SPE Cartridges MCX, 30 μm, Waters), concentrated by vacuum centrifugation, and reconstituted in 40 μL of 0.1% (v/v) formic acid. The peptide content was estimated by UV light spectral density at 280 nm. For DIA experiments, iRT (indexed retention time) calibration peptides were spiked into the sample.

#### 2.6.3 Liquid Chromatography–Tandem Mass Spectrometry (DIA Mode) Analysis

The peptides from each sample were analyzed by an OrbitrapTM AstralTM mass spectrometer (Thermo Scientific) connected to a Vanquish Neo liquid chromatography system (Thermo Scientific) in the data‐independent acquisition (DIA) mode. Precursor Ions were scanned at a mass range of 380‐980 m/z, MS1 resolution was 240,000 at 200 m/z; normalized AGC Target: 500%, maximum IT: 5 ms. 299 windows were set for DIA mode in MS2 scanning, Isolation Window: 2 m/z, HCD Collision Energy: 25ev, Normalized AGC Target: 500%, Maximum IT: 3 ms.

#### 2.6.4 Data Processing and Protein Identification

DIA data were analyzed with DIA‐NN 1.8.1. Bioinformatic analysis was performed for functional annotation and interpretation. Hierarchical clustering, subcellular localization, and protein domain prediction were conducted using Cluster 3.0, CELLO, and InterProScan, respectively. GO and KEGG annotations and enrichment analyses were performed to explore biological functions. GSEA, PPI network construction, PCA, and WGCNA were carried out to identify key proteins and modules. Transcription factors and kinases were annotated using corresponding public databases.

### 2.7 Western Blot Analysis

Prostate tissues were lysed in RIPA buffer containing PMSF, and protein concentrations were determined using a bicinchoninic acid assay. Equal amounts of protein (15 μg per lane) were separated on 8% SDS‐PAGE gels and transferred onto 0.45 μm polyvinylidene fluoride (PVDF) membranes. After blocking with 5% non‐fat milk, the membranes were incubated overnight at 4°C with antibodies against ALDH1B1 (Proteintech, 85,426–2‐RR) and β‐actin, which served as the loading control, followed by the corresponding secondary antibodies. Protein bands were detected using enhanced chemiluminescence with a Tanon 5200 imaging system and quantified by densitometric analysis using ImageJ software (Wang et al. 2025; Du et al. 2019; Guan et al. 2025).

### 2.8 Immunohistochemical Staining of GPX4

Prostate tissues were fixed in 4% paraformaldehyde, paraffin‐embedded, and sectioned at a thickness of 2–3 μm. Sections from eight samples per group were deparaffinized and rehydrated, followed by heat‐induced antigen retrieval in EDTA buffer at pH 9.0. After blocking endogenous peroxidase activity and nonspecific binding, the sections were incubated overnight at 4°C with a rabbit anti‐GPX4 antibody at a 1:200 dilution (Affinity Biosciences, DF6701), followed by HRP‐conjugated goat anti‐rabbit IgG at a 1:500 dilution (SeraCare, 5220‐0336). Staining was visualized with 3,3′‐diaminobenzidine, counterstained with hematoxylin, dehydrated, and mounted. Images were acquired at 200× magnification, and microscopic fields were selected using a blinded method (Guan et al. 2025; Chen et al. 2024).

## 3 Results

### 3.1 Identification of Major Bioactive Components in QLZTF (UPLC–MS Analysis)

The chemical composition of QLZTF was analyzed using UPLC‐MS/MS. A total of 36 components absorbed into blood were identified, including 8 from Linderae Radix, 1 from Persicae Semen, 6 from Carthami Flos, 3 from Sargentodoxae Caulis, 2 from Corydalis Rhizoma, 7 from Polygoni Cuspidati Rhizoma et Radix, and 10 from Bupleuri Radix. Representative compounds included linderane, linderol, bornyl acetate, kaempferol 3‐O‐β‐D‐glucuronide, procyanidin A2, procyanidin B1, hydroxyisogermafurenolide, lindestrene from Linderae Radix; amygdalin from Persicae Semen; carthamin yellow A, hydroxysafflor yellow A, kaempferol‐3‐O‐rutinoside, rutin, luteolin‐7‐O‐β‐D‐glucopyranoside, baicalin from Carthami Flos; chlorogenic acid, chrysophanol, emodin from Sargentodoxae Caulis; dehydrocorydaline, berberine from Corydalis Rhizoma; quercetin‐3‐O‐β‐D‐glucoside, polydatin, polygoside A, Ω‐hydroxyemodin‐8‐methyl ether, chrysophanol, 6‐hydroxyalocemodin, emodin from Polygoni Cuspidati Rhizoma et Radix; and 6‐methoxy‐7‐hydroxycoumarin, scoparone, 6,7,8‐trimethoxycoumarin, saikosaponin F, C, A, D, 3′‐O‐acetylsaikosaponin A, 23‐O‐acetylsaikosaponin D, saikosaponin E from Bupleuri Radix. The total ion chromatogram is shown in Figure 1, and detailed identification is listed in Table 1.

**FIGURE 1:**
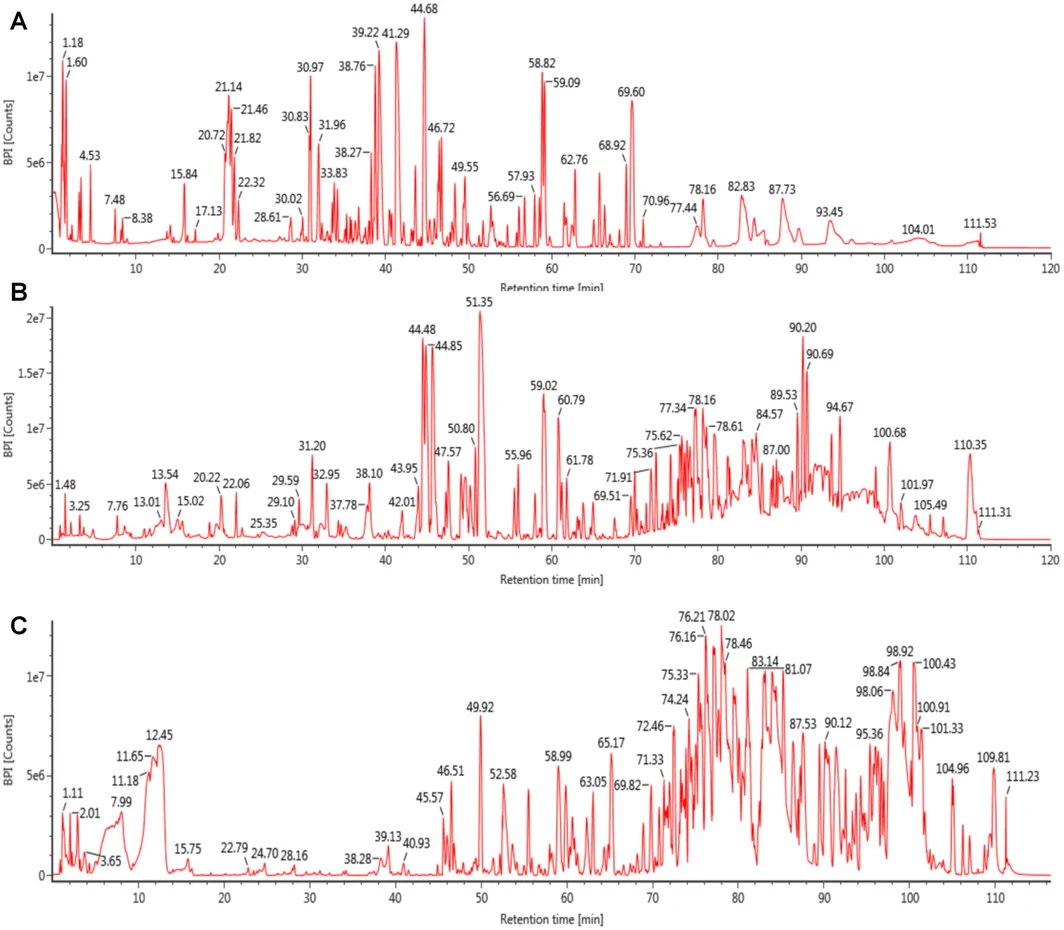
UPLC‐MS total ion chromatogram of QLZTF. (A) QLZTF extract; (B) Drug‐containing serum; (C) Blank serum.

**TABLE 1:** Identification of chemical components absorbed into blood from QLZTF extract (positive/negative ion mode).

No.	tR/min	Compound	Molecular formula	m/z	Error/ppm	Adducts	Source
1	1.2	Linderane	C15H18O2	253.1189	−4	+Na	Prototype
2	1.71	Linderol	C15H18O2	253.1191	−3.3	+Na	Prototype
3	3.53	Carthamin Yellow A	C27H30O15	595.1654	−0.6	+H	Prototype
4	7.68	Bornyl Acetate	C12H20O2	219.1345	−4.6	+Na	Prototype
5	9.51	Quercetin‐3‐O‐β‐D‐glucoside	C21H20O12	463.0887	1.2	−H	Prototype
6	10.01	Morroniside	C17H26O11	407.1533	−3.7	+H	Prototype
7	10.72	Hydroxysafflor Yellow A	C27H32O16	611.163	2	−H	Prototype
8	18.67	6‐Methoxy‐7‐hydroxycoumarin	C10H8O4	193.0502	3.6	+H	Prototype
9	19.02	Amygdalin	C20H27NO11	480.1482	1.3	+Na, +H	Prototype
10	19.83	Chlorogenic Acid	C16H18O9	377.085	1.8	+Na	Prototype
11	22.02	Polydatin	C20H22O8	413.1217	2.5	+Na	Prototype
12	27.91	Polygoside A	C14H12O4	243.0658	−1.9	−H	Metabolite
13	28.01	Kaempferol‐3‐O‐rutinoside	C27H30O15	595.1675	2.9	+H	Prototype
14	28.41	Rutin	C27H30O16	633.1468	6.7	+Na, +H	Prototype
15	31.67	Kaempferol 3‐O‐β‐D‐glucuronide	C21H18O12	463.0878	1.5	+H, +Na	Prototype
16	31.77	Scoparone	C11H10O4	205.0492	−7	−H	Prototype
17	32.45	Dehydrocorydaline	C21H23NO4	354.17	0	+H	Prototype
18	34.51	Luteolin‐7‐O‐β‐D‐glucopyranoside	C21H20O11	449.1094	3.5	+H	Prototype
19	34.82	Procyanidin A2	C30H24O12	577.1313	−4.8	+H	Prototype
20	35.41	Baicalin	C21H18O11	447.0931	2	+H	Prototype
21	39.48	Berberine	C20H17NO4	336.1221	−2.8	+H	Prototype
22	39.9	6,7,8‐Trimethoxycoumarin	C12H12O5	235.0599	−5.6	−H	Metabolite
23	42	Ω‐Hydroxyemodin‐8‐methyl ether	C16H12O6	299.0578	5.6	−H	Prototype
24	48.15	1,8‐Dihydroxy‐3‐methylanthraquinone (Chrysophanol)	C15H10O4	253.0507	0.1	−H	Metabolite
25	49.96	6‐Hydroxyalocemodin	C15H10O6	285.04	−1.5	−H	Prototype
26	52.32	Procyanidin B1	C30H26O12	577.1362	1.9	−H	Metabolite
27	53.47	Hydroxyisogermafurenolide	C15H20O3	247.133	−4	−H	Prototype
28	56.58	Saikosaponin F	C48H80O17	951.53	1.3	+Na	Prototype
29	57.82	Saikosaponin C	C48H78O17	949.514	0.9	+Na	Prototype
30	61.14	Lindestrene	C15H16O4	259.0965	−4	−H	Prototype
31	62.76	Saikosaponin A	C42H68O13	803.4556	0.4	+Na	Prototype
32	65.73	Saikosaponin D	C42H68O13	803.4553	0.1	+Na	Prototype
33	68.96	3′‐O‐Acetylsaikosaponin A	C44H70O14	845.4672	1.7	+Na	Prototype
34	69.52	Emodin	C15H10O5	269.0447	−3	−H	Prototype
35	71.03	23‐O‐Acetylsaikosaponin D	C44H70O14	845.4675	2	+Na	Prototype
36	71.04	Saikosaponin E	C42H68O12	787.4626	3	+Na	Prototype

### 3.2 In Vivo Therapeutic Efficacy of the QLZTF in CP/CPPS Rats

#### 3.2.1 Overall Experimental Design and Basic Indicators

The animal experimental protocol is illustrated in Figure 2A, and the schematic diagram of the surgical procedure is shown in Figure 2B–E. As shown in Figure 2F, rats in the Control, Model, Qianliekang, and QLZTF low‐ and high‐dose groups all exhibited a stable linear growth trend. At week 0, the initial body weight was approximately 360 g in all groups; by week 4, the body weight reached approximately 510 g in each group. The growth curves of the five groups were nearly overlapping, with no statistically significant differences observed.

**FIGURE 2:**
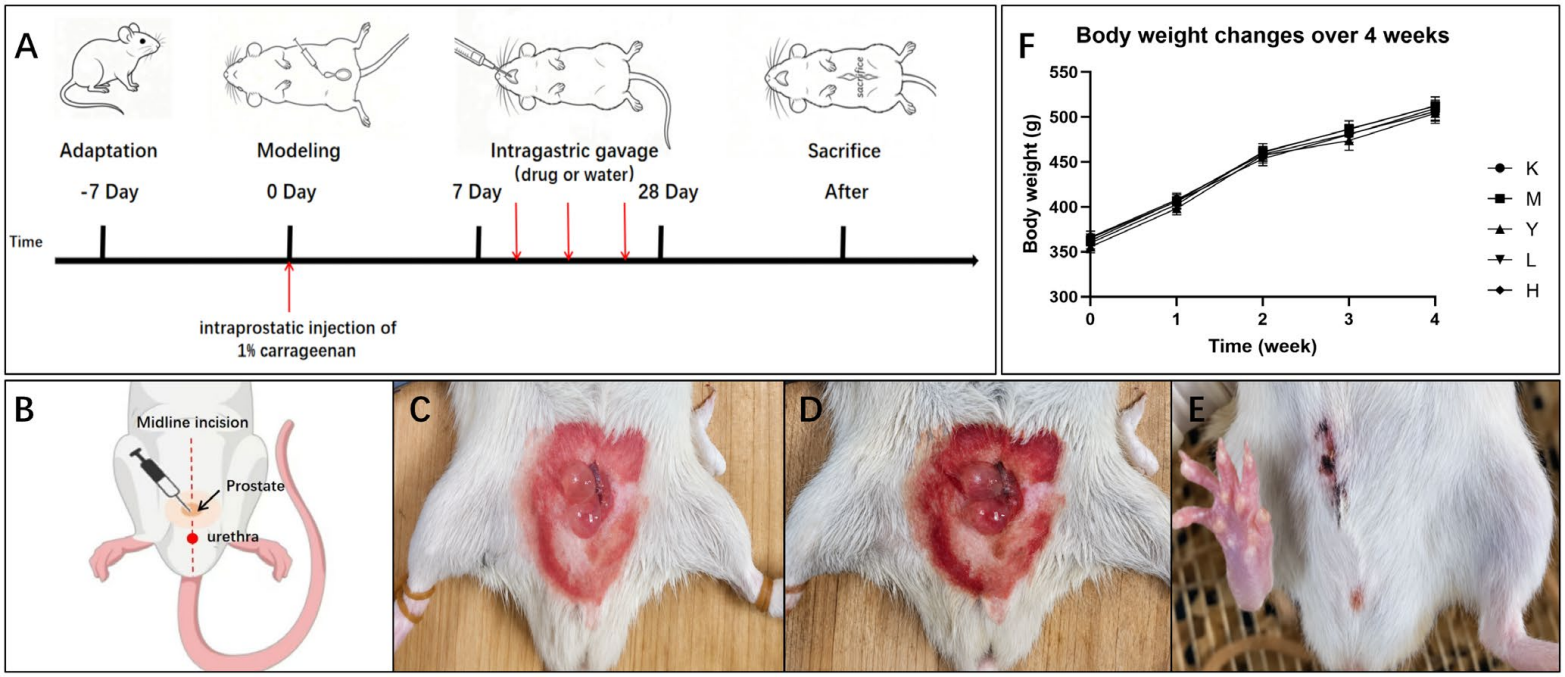
Schematic diagrams of the animal experiment protocol and the surgical procedure for establishing a rat model of chronic prostatitis. (A) Simple schematic diagram of the animal experiment protocol. (B) A longitudinal incision is made along the midline of the abdomen, and the abdominal cavity is opened layer by layer. (C) The bladder is gently retracted to one side with forceps to expose the ventral prostate lobes located dorsally to the bladder. (D) Using a microsyringe, 50 μL of 1% carrageenan solution is administered into each of the bilateral ventral prostate lobes (100 μL in total). (E) The muscle and skin are sutured layer by layer. (F) Body weight changes over the 5‐week experimental period: Groups are color‐coded as indicated (K: Control; M: Model; Y: Qianliekang; A: QLZTF low‐dose; B: QLZTF high‐dose).

#### 3.2.2 QLZTF Reduced the Expression of Proinflammatory Cytokine Levels in Prostate Tissue

Since proinflammatory cytokines are direct biomarkers of inflammation, three key factors were utilized to assess inflammatory degrees in each group. As shown in Table 2, the expression of TNF‐α, IL‐6, and IL‐1β remarkably enhanced respectively in the model group compared with those in the normal control group. However, QLZTF effectively inhibited the upregulation of these proinflammatory cytokines.

**TABLE 2:** Pharmacodynamic indicators of QLZTF.

Group	Dose (g/kg)	TNF‐α (pg/mL)	IL‐6 (pg/mL)	IL‐1β (pg/mL)
Control (*n* = 8)	—	95.34 ± 8.64	52.35 ± 8.53	66.54 ± 9.32
Model (*n* = 8)	—	160.85 ± 13.72**	129.46 ± 9.32**	150.51 ± 12.65**
Qianliekang (*n* = 8)	0.5	106.63 ± 11.16^#^	101.24 ± 8.31^#^	128.42 ± 10.76^#^
QLZTF low‐dose (*n* = 8)	1.7	146.45 ± 6.57^#^	119.35 ± 14.78	137.83 ± 9.35^#^
QLZTF high‐dose (*n* = 8)	6.8	125.68 ± 12.72^#^	97.53 ± 11.49^#^	105.29 ± 11.42^##^

*Note:* Compared with control group, *p < 0.05, **p < 0.01; compared with model group, ^*#*^ *p* < 0.05, ^*##*^ *p* < 0.01.

#### 3.2.3 Histopathological Findings of Prostate Tissue Using H&E and IHC Staining

Histopathological results from H&E staining (Figure 3) showed that the control group exhibited intact prostatic tissue structure with clear boundaries, regular glandular lumina, and neatly arranged glandular epithelial cells, accompanied by a small amount of inflammatory cell infiltration in the interstitium. In the model control group, compared with the control group, the H&E staining results of rat prostate tissue showed obvious changes, including vascular dilation and congestion, reduced luminal secretions, significantly narrowed acinar lumina, papillary hyperplasia of acinar epithelium, loose stromal edema with fibrosis, and extensive lymphocyte infiltration around the glandular stroma. In the Qianliekang group, the prostatic tissue showed enlarged and irregular glandular lumina, disordered arrangement of glandular epithelial cells, and reduced lymphocyte infiltration around the glandular stroma. In the QLZTF group, most glandular lumina maintained an intact structure, although the glandular epithelial cells were irregularly arranged with basement membrane invaginating into the lumina, and a small amount of inflammatory cell infiltration was observed in the interstitium, as shown in Figure 3.

**FIGURE 3:**
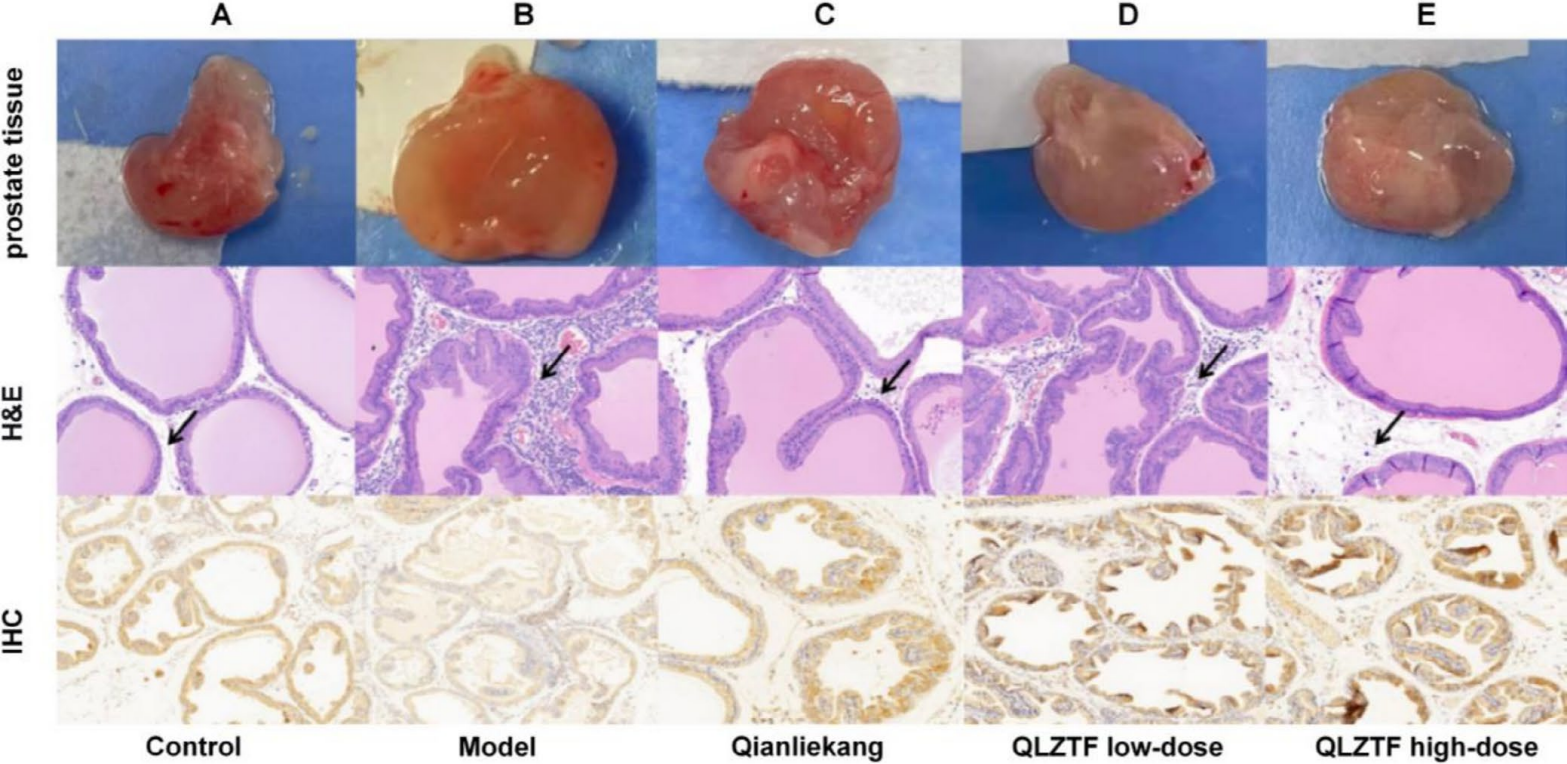
H&E and IHC Staining of rat prostate tissue in each group (×200). (A) Control group; (B) Model control group; (C) Qianliekang group; (D) Low‐dose QLZTF group; (E) High‐dose QLZTF group.

To further verify the involvement of ferroptosis in the therapeutic effects of QLZTF, we examined the expression of GPX4, a key ferroptosis marker, in prostate tissues using immunohistochemistry (IHC). Immunohistochemical staining revealed distinct differences in GPX4 expression across the experimental groups. In the control group, strong GPX4 immunoreactivity was observed predominantly in the glandular epithelial cells. In contrast, GPX4 staining was markedly reduced in the model group. Treatment with QLZTF restored GPX4 expression, with increased immunostaining intensity observed in both low‐dose and high‐dose groups. The Qianliekang group showed a similar trend (Figure 3). These results demonstrate that QLZTF treatment effectively restores GPX4 expression in prostate tissues of CP/CPPS rats.

#### 3.2.4 Pain Response Measurement (von Frey Filaments)

Before treatment (Week 0), the perineal pain response frequency of rats in each group was measured using von Frey filaments. As shown in Figure 4A, compared with the Control group, the Model, Qianliekang, and QLZTF low‐ and high‐dose groups all exhibited significantly increased response frequencies under 4 g stimulation (*p* < 0.01), indicating heightened pain sensitivity after model induction.

**FIGURE 4:**
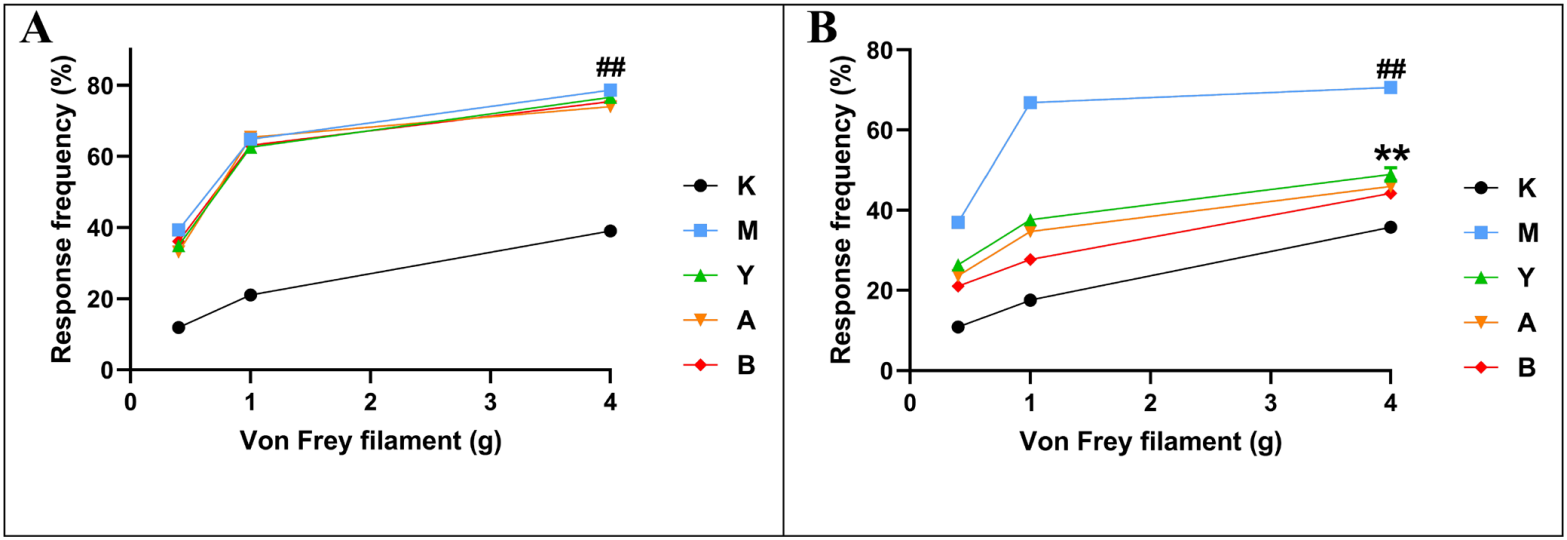
Measurement of pain behavior. Pain response frequency of each group under stimulation with von Frey filaments at forces of 0.4 g, 1.0 g, and 4.0 g at week 0 (A) and week 4 (B). Data are presented as mean ± SD. Compared with the control group, ^*#*^ *p *< 0.05, ^*##*^ *p* < 0.01; compared with the model group, *p < 0.05, **p < 0.01.

After 4 weeks of continuous administration, as shown in Figure 4B, compared with the Control group, the Model group still showed a significantly increased response frequency (*p* < 0.01). Compared with the Model group, the Qianliekang group and both QLZTF dose groups displayed significantly reduced response frequencies (*p* < 0.01). Among these, the QLZTF high‐dose group showed a response frequency of approximately 45%, which was closest to that of the Control group (approximately 35%). No significant difference was observed between the QLZTF low‐dose and high‐dose groups (*p* > 0.05). These results indicate that QLZTF effectively alleviates pelvic pain in rats with chronic prostatitis, with comparable efficacy between the high and low doses.

### 3.3 Proteomics Reveals That QLZTF Upregulates BCAA Metabolism and CoA Biosynthesis Pathways to Inhibit Ferroptosis

The technique of Label‐free proteomics was applied to investigate the protein expression alterations in the prostate. In order to investigate the overall changes in the prostate among different groups, multivariate statistical analysis was performed on the prostate proteomics data. A total of 236 differentially expressed proteins were identified, and the separation trends of prostate proteomics profiles among experimental groups were evident in the principal component analysis (PCA) score plots, as shown in Figure 5A. It is worth noting that the effect of the QLZTF group includes the Qianliekang group, while they both are different from the model group and the control group. Proteins with FC > 1.5 or < 0.67 and *p* < 0.05 were considered as screening criteria for differentially expressed proteins (DEPs). In comparison with the model group, 241 (upregulation) and 642 (downregulation) DEPs were identified in the QLZTF group (Figure 5B). The DEPs were systematically categorized according to their MF, CC, and BP using the GO database (Figure 5C). 175 pathways were enriched in KEGG pathway analysis, and the top 20 associated with CP/CPPS were shown in Figure 5D. KEGG analysis revealed that 140 downregulated and eight upregulated proteins were enriched in the model group. KEGG analysis was performed separately for these DEPs. Fisher's exact test (*p* < 0.05) revealed that DEPs were significantly enriched in the Pantothenate and CoA Biosynthesis and Valine, leucine and isoleucine degradation pathways. BCAT2, ALDH1B1, and ALDH2 were upregulated. KEGG pathway enrichment analysis further identified multiple additional pathways that have been implicated in CP/CPPS pathogenesis. The protein processing in endoplasmic reticulum pathway was significantly enriched in the QLZTF group (*p* < 0.001, GeneRatio = 10/108), involving 10 differentially expressed proteins including Sec61b, Lman1, Tusc3, Tram1, Ostc, Sec31a, Derl2, Sec24d, Rrbp1, and Herpud1. The tryptophan metabolism pathway was also significantly altered (*p* = 0.003, GeneRatio = 4/108), involving Aldh1b1, Gcdh, Dhtkd1, and Aldh2. Additionally, the fatty acid degradation pathway was significantly enriched (*p* = 0.003, GeneRatio = 4/108), involving Gcdh, Adh1, Aldh1b1, and Aldh2. Among the upregulated DEPs, BCAT2, ALDH1B1, and ALDH2 were the main proteins involved in the Pantothenate and CoA biosynthesis and Valine, leucine, and isoleucine degradation pathways, and the expression levels were significantly upregulated after treatment with QLZTF. Notably, key pathways were significantly associated with ferroptosis and oxidative stress in the QLZTF treatment group.

**FIGURE 5:**
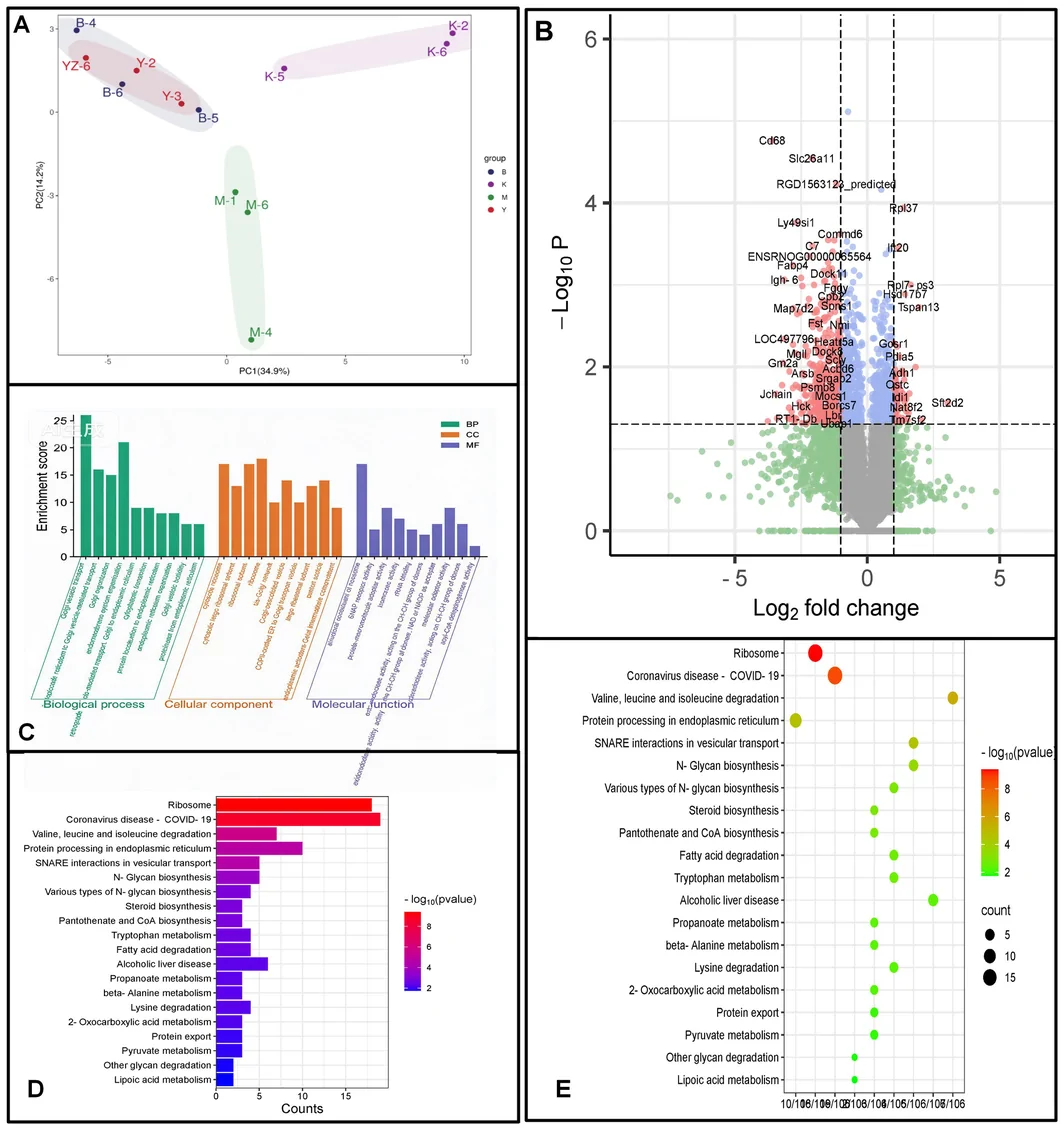
Assaying key DEPs that QLZTF alters the proteomics profile of CP/CPPS rats through proteomics. (A) PCA of the different abundance proteins among QLZTF, model, control, and Qianliekang groups. Dot plot representing PCA scores of proteomics among four groups; *n* = 3, *n* represents the detection number of each group. (B) Volcano plot for the QLZTF group vs. the model group. (C) Top 10 GO terms of the differently abundant proteins. (D‐E) Top 20 KEGG pathways of the differently abundant proteins.

### 3.4 Metabolomics Confirms the Involvement of BCAA and CoA Biosynthesis Pathways in QLZTF's Therapeutic Effects

Metabolomics analysis was performed on prostate samples. Principal components analysis (PCA) indicated a separation between the QLZTF group and both the normal and model groups (Figure 6A). OPLS‐DA analyses of prostate samples metabolites between the QLZTF group and the model group were conducted (*R* ^2^ = 0.773, *Q* ^2^ = 0.373), demonstrating significant differences in prostate samples metabolites across the four groups (Figure 6B). Overall, 15,427 metabolites were identified in ESI+ and ESI‐ modes, among which 1449 metabolites could be unambiguously matched in the database. The DMs with fold change (FC) ≥ 2 or ≤ 0.5, and variable importance in projection (VIP) ≥ 1 were considered as differential markers. A total of 436 differential metabolites were identified between the QLZTF group and the model group. Among these, volcano plot analysis further identified 84 significantly upregulated and 41 significantly downregulated metabolites in the QLZTF group compared with the model group (Figure 6C).

**FIGURE 6:**
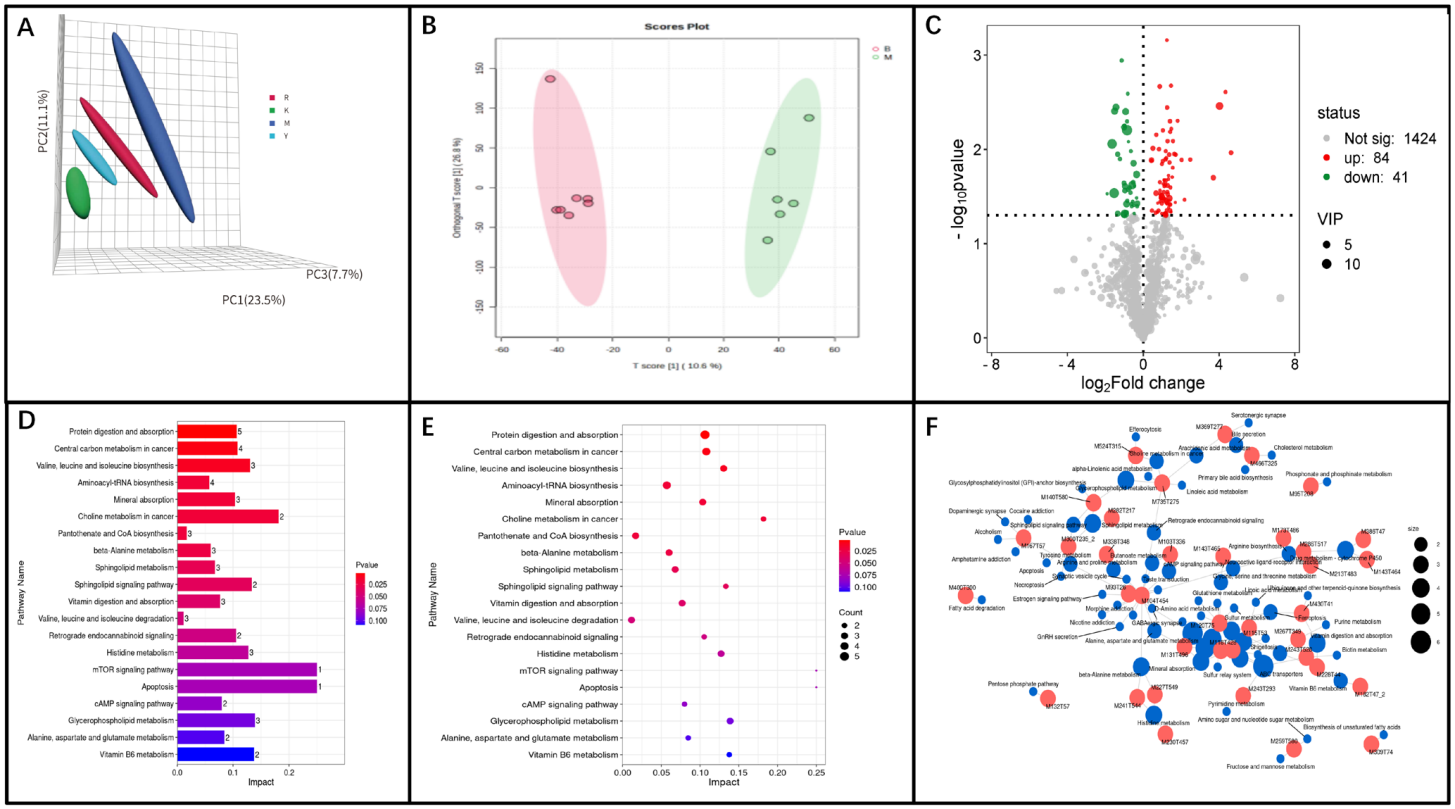
Assaying key metabolic pathways for improving metabolic abnormalities in rats study through metabolomics. (A) 3D PCA analyses of prostate tissue metabolites. (B) OPLS‐DA analyses of prostate metabolites. (C) Volcano plot for metabolite analysis in prostate tissues from the model group and the QLZTF group. (D‐F) KEGG pathway analysis of differentially abundant metabolites. *p < 0.05, **p < 0.01, *p < 0.001 vs. the model group, *n* = 6, n represents the number of sequencing samples in each group.

KEGG pathway enrichment analysis was performed on the differential metabolites to identify the major metabolic pathways. Bar and bubble plots were generated based on *p*‐values and rich factors (Figure 6D,E). Integrated metabolomic analysis revealed that both the Pantothenate and CoA biosynthesis pathway and the Valine, leucine, and isoleucine degradation (BCAA) pathway were significantly enriched in the metabolomic KEGG analysis. Therefore, these two pathways were identified as key pathways for further investigation (Table 3). Metabolomic KEGG enrichment analysis also revealed two additional pathways closely associated with CP/CPPS. The sphingolipid metabolism pathway was significantly enriched (*p* = 0.03, Hits = 3). The glycerophospholipid metabolism pathway was also enriched in the QLZTF‐treated group (*p* = 0.05, Hits = 3). Both pathways have been reported to be involved in chronic inflammation‐induced prostate pathology and ferroptosis.

**TABLE 3:** KEGG pathway enrichment analysis of differential metabolites.

KEGG pathway	Total	Hits	*p*	Impact
Protein digestion and absorption	47	5	0.002	0.106
Central carbon metabolism in cancer	37	4	0.005	0.108
Valine, leucine and isoleucine biosynthesis	23	3	0.010	0.130
Aminoacyl‐tRNA biosynthesis	52	4	0.018	0.057
Mineral absorption	29	3	0.018	0.103
Choline metabolism in cancer	11	2	0.018	0.182
Pantothenate and CoA biosynthesis	30	3	0.020	0.017
Beta‐Alanine metabolism	32	3	0.024	0.060
Sphingolipid metabolism	35	3	0.030	0.068
Sphingolipid signaling pathway	15	2	0.034	0.133
Vitamin digestion and absorption	39	3	0.039	0.077
Valine, leucine and isoleucine degradation	42	3	0.048	0.012
Retrograde endocannabinoid signaling	19	2	0.032	0.105
Histidine metabolism	47	3	0.063	0.127
mTOR signaling pathway	4	1	0.076	0.250
Apoptosis	4	1	0.026	0.250
cAMP signaling pathway	25	2	0.035	0.080
Glycerophospholipid metabolism	56	3	0.050	0.139
Alanine, aspartate and glutamate metabolism	28	2	0.013	0.085
Vitamin B6 metabolism	29	2	0.109	0.138

### 3.5 Validation of Key Proteins by Western Blot

To validate the reliability of the proteomic profiling results, we performed Western blot analysis to detect the protein expression levels of ALDH1B1 in prostate tissues across four experimental groups: control (K), model (M), Qianliekang (Y), and QLZTF high‐dose (B).

As shown in Figure 7A, the protein band of ALDH1B1 (57 kDa) was clearly detected in all groups. The band intensity of ALDH1B1 was highest in the control group (K) and lowest in the model group (M), while the Qianliekang group (Y) and QLZTF high‐dose group (B) showed intermediate expression levels. β‐Actin (43 kDa) served as the loading control and exhibited consistent expression across all groups, confirming equal protein loading.

**FIGURE 7:**
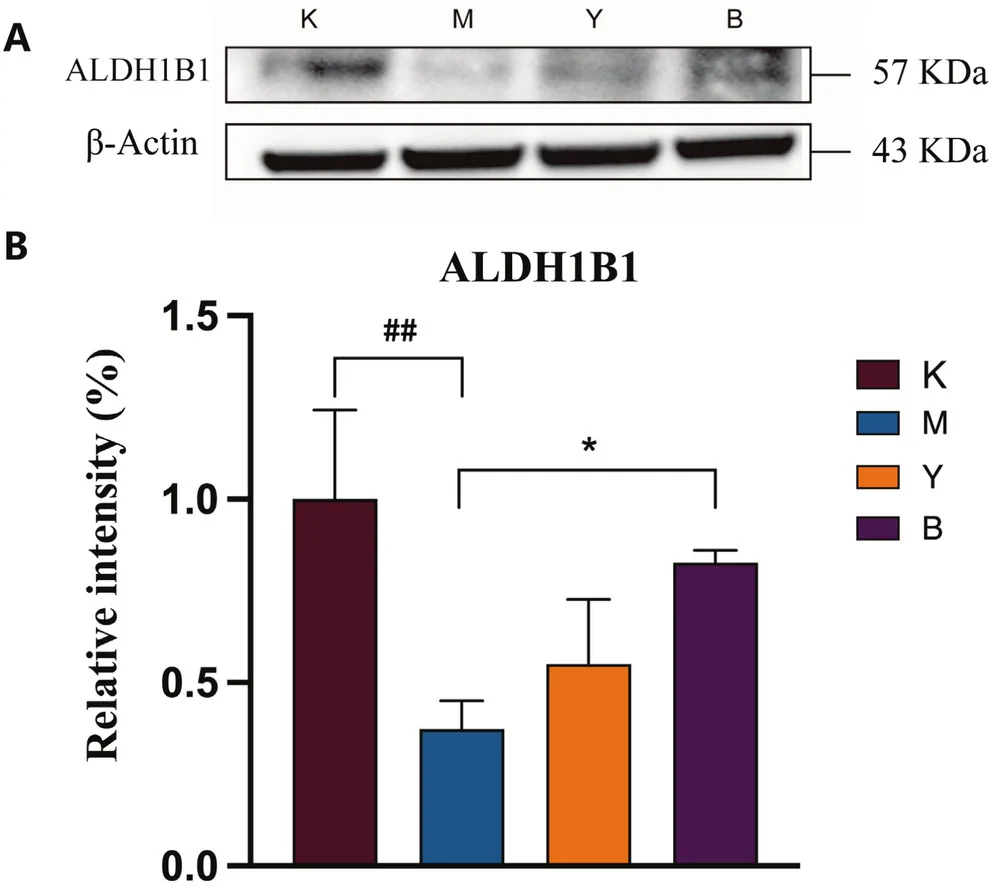
(A) Representative Western blot images showing the protein expression levels of ALDH1B1 (57 KDa) and β‐Actin (43 KDa) as a loading control across four experimental groups: K (control group, red), M (model group, blue), Y (Qianliekang, orange), and B (QLZTF high‐dose, purple). (B) Quantitative analysis of ALDH1B1 relative expression levels normalized to β‐Actin. Data are presented as mean ± standard deviation. Statistical significance is indicated as follows: ^*##*^ *P* < 0.01 vs. group K; *p < 0.05 between groups M and B.

Quantitative analysis revealed that ALDH1B1 expression was significantly decreased in the model group compared with the control group (*p* < 0.01, Figure 7B). Treatment with QLZTF (B) and Qianliekang (Y) both upregulated ALDH1B1 expression, with the QLZTF high‐dose group showing higher expression than the Qianliekang group (*p* < 0.05, Figure 7B). These results confirm that QLZTF treatment effectively upregulates ALDH1B1 expression in prostate tissues of CP/CPPS rats, consistent with our proteomic findings.

## 4 Discussion and Conclusions

In this study, we integrated proteomics and metabolomics to systematically investigate the therapeutic mechanism of QLZTF in chronic prostatitis/chronic pelvic pain syndrome (CP/CPPS). Proteomics revealed 241 upregulated and 642 downregulated differentially expressed proteins in the QLZTF group compared to the model group, with significant upregulation of BCAT2, ALDH1B1, and ALDH2. Metabolomics further demonstrated substantial alterations in the prostate metabolic profile upon QLZTF intervention, identifying 436 differential metabolites. Notably, the Pantothenate and CoA Biosynthesis pathway and the Valine, leucine and isoleucine degradation (Branched‐Chain Amino Acid, BCAA) pathway were significantly enriched in both proteomic and metabolomic KEGG analyses, indicating that these two pathways serve as core metabolic hubs mediating the therapeutic effects of QLZTF.

What is the relationship between the Pantothenate/CoA biosynthesis and BCAA degradation pathways and CP/CPPS. First, BCAA (valine, leucine, isoleucine) are not only substrates for protein synthesis but also participate in regulating energy metabolism, oxidative stress, and immuno‐inflammatory responses (Peng et al. 2021). Recent studies have directly demonstrated that dysregulated BCAA metabolism promotes ferroptosis, thereby exacerbating tissue damage and inflammatory responses (Bonvini et al. 2018); conversely, promoting BCAA metabolism effectively inhibits ferroptosis and alleviates pathological damage (Li et al. 2023). Furthermore, a BCAA metabolite, β‐aminoisobutyric acid, has been shown to activate the AMPK/Nrf‐2 pathway, upregulate GPX4 and SLC7A11 expression, and thereby inhibit ferroptosis (Zhang et al. 2023). Second, pantothenate (vitamin B5) is a precursor for coenzyme A (CoA) synthesis. CoA, as an essential cofactor for over 100 enzymatic reactions, is deeply involved in energy metabolism, lipid metabolism, and the synthesis and detoxification of inflammatory mediators (Chen et al. 2024). In the local chronic inflammatory microenvironment of the prostate, impaired energy metabolism and increased oxidative stress are key drivers of persistent inflammation, fibrosis, and pain sensitization. Therefore, the coordinated dysregulation of these two pathways likely promotes ferroptosis, thereby exacerbating the pathological progression of CP/CPPS.

After establishing the association between these two key pathways and CP/CPPS, our study further elucidates the specific molecular mechanisms by which QLZTF inhibits ferroptosis. Our data indicate that the QLZTF‐upregulated BCAT2, ALDH1B1, and ALDH2 are critical anti‐ferroptotic effector proteins. The core initiating elements of ferroptosis include reactive oxygen species (ROS), oxidizable lipids, and lipid peroxidation, with ROS serving as the primary initiator. BCAT2 inhibits ferroptosis and mitigates oxidative stress by modulating glutamate‐cystine exchange to promote cysteine consumption, thereby reducing ROS accumulation (Wang, Zhang, et al. 2021). ALDH1B1 inhibits NOX1‐mediated ferroptosis through the catalytic oxidation of toxic aldehydes (e.g., 4‐hydroxynonenal) to carboxylic acids (Chen et al. 2022). ALDH2 exerts anti‐ferroptotic effects via multiple mechanisms: detoxification of toxic aldehydes, NADH generation, pH regulation, and promotion of lipid droplet formation (Shan et al. 2024); these lipid droplets act as lipid reservoirs to sequester oxidizable lipids, thereby interrupting the chain reaction of lipid peroxidation (Danielli et al. 2023). These findings are highly consistent with the pioneering report by Lin et al. demonstrating the involvement of ferroptosis in chronic prostatitis progression (Lin et al. 2022).

To further elucidate the molecular mechanisms by which QLZTF modulates metabolic reprogramming, we screened multiple KEGG pathways closely associated with CP/CPPS. Regarding the protein processing in the endoplasmic reticulum pathway, previous studies have indicated that pathological microenvironment remodeling in CP/CPPS involves local inflammatory responses, immune cell infiltration, and oxidative stress, which are closely associated with endoplasmic reticulum dysfunction (Zhang et al. 2024). For the tryptophan metabolism pathway, Zhang et al. recently reported that the tryptophan pathway is the most significantly altered metabolic pathway associated with distinct CP/CPPS phenotypes, and its dysregulation is closely linked to the neuropathological mechanisms underlying chronic pelvic pain syndrome (Zhang et al. 2025). In terms of the fatty acid degradation pathway, studies have demonstrated that CXCR4 regulates polyunsaturated fatty acid metabolism via the PPARγ/Fads2 axis, and downregulation of CXCR4 effectively alleviates inflammatory cell infiltration and oxidative stress injury in experimental prostatitis (Zhang et al. 2024). At the metabolomic level, Li et al. employed LC‐MS‐based untargeted metabolomics and confirmed that sphingolipid metabolism is the most significantly altered pathway in patients with chronic inflammation‐associated prostate pathology, and that glycerophospholipid metabolism is also substantially dysregulated, a pathway intimately linked to ferroptosis (Li, Wu, et al. 2024). Beyond these specific metabolic and inflammatory pathways, emerging evidence has highlighted the involvement of broader regulatory mechanisms in chronic inflammatory diseases. Posttranslational modifications such as SUMOylation have been shown to regulate tumorigenesis and disease progression through modulating protein function and stability (Wu et al. 2026). Concurrently, the cytokine‐epigenetic axis, which bridges inflammatory signals with epigenetic reprogramming, has emerged as a critical paradigm in the pathogenesis of inflammatory disorders (Luo et al. 2026). It is plausible that QLZTF, as a multicomponent formula, may exert its therapeutic effects not only through metabolic pathway regulation but also via modulation of these higher‐order regulatory networks, although this hypothesis requires further experimental validation. The systematic enrichment of these multiple pathways further supports that QLZTF may exert synergistic anti‐inflammatory and anti‐oxidative stress effects by modulating lipid metabolism and endoplasmic reticulum stress pathways.

Consistent with these pathway alterations, the pharmacodynamic results of this study showed that QLZTF significantly reduced the levels of inflammatory cytokines (TNF‐α, IL‐6, and IL‐1β) and pain response in rats with chronic prostatitis while alleviating pathological damage in prostate tissue. These phenotypic improvements further support the conclusion that QLZTF exerts its therapeutic effects by inhibiting ferroptosis and reprogramming metabolism.

To further support the pharmacodynamic basis of QLZTF, we reviewed the literature on some of its blood‐absorbed active components. Hydroxysafflor Yellow A (HSYA) from Carthami Flos exhibits anti‐inflammatory and antioxidant activities and can inhibit inflammatory responses via the Toll‐like receptor 4 pathway (Wang, Xiao, et al. 2021). Saikosaponin D from Bupleuri Radix has been shown to suppress the NF‐κB pathway, alleviating pelvic pain and prostatic inflammation in mice with chronic prostatitis (Wang et al. 2026). Furthermore, additional literature analysis suggests that hydroxysafflor yellow A from Carthami Flos can inhibit ferroptosis by activating the HIF‐1α/SLC7A11/GPX4 signaling pathway (Ge et al. 2023); saikosaponin A from Bupleuri Radix has also been reported to regulate key ferroptosis‐related proteins such as GPX4 and ACSL4 (Li, Liu, et al. 2024). These findings provide component‐level evidence supporting the anti‐ferroptotic effect observed in our study. These findings suggest that multiple active components in QLZTF may act synergistically to exert anti‐inflammatory and analgesic effects, providing literature‐based evidence for its therapeutic efficacy in CP/CPPS.

In conclusion, this study demonstrates for the first time that QLZTF synergistically regulates the Pantothenate/CoA Biosynthesis and BCAA degradation pathways to activate the BCAT2/ALDHs axis, effectively inhibiting ferroptosis and thereby alleviating inflammation, fibrosis, and pain in CP/CPPS. The elucidation of this “pathway‐metabolism‐ferroptosis” mechanism not only provides an experimental basis for the clinical application of QLZTF but also serves as a paradigm for multiomics research on traditional Chinese medicine compounds. Future studies employing techniques such as gene knockout/overexpression and cellular thermal shift assay (CETSA) are warranted to further validate the direct interactions between specific active components of QLZTF and BCAT2, ALDH1B1, and ALDH2.

## Author Contributions

**Lidan Dong:** writing – review and editing. **Linhan Chang:** visualization, writing – original draft. **Caijia Song:** validation. **Jialin Yan:** methodology. **Tiantong Shen:** data curation. **Zhen Ding:** data curation. **Yunyu Liu:** data curation. **Chenglong Zheng:** data curation. **Yanfei Zheng:** conceptualization. **Qi Wang:** conceptualization, supervision.

## Funding

This work was supported by the High‐level Key Discipline of Traditional Chinese Medicine‐Constitution of Traditional Chinese Medicine, National Administration of Traditional Chinese Medicine (Grant No. zyyzdxk‐2023251); Fundamental Research Funds for the Central Universities, Beijing University of Chinese Medicine (2022‐JYB‐XJSJJ037, 2025‐JYB‐JBGS‐030).

## Disclosure

The authors confirm that no generative AI tools were used in the preparation of this manuscript.

## Consent

The authors have nothing to report.

## Conflicts of Interest

The authors declare no conflicts of interest.

## Data Availability

The raw data supporting the conclusions of this article will be made available by the authors, without undue reservation.
